# CFD Simulations of Microreactors for the Hydrolysis of Cellobiose to Glucose by β-Glucosidase Enzyme

**DOI:** 10.3390/mi11090790

**Published:** 2020-08-21

**Authors:** Virginia Venezia, Valeria Califano, Giulio Pota, Aniello Costantini, Gianluca Landi, Almerinda Di Benedetto

**Affiliations:** 1 Dipartimento di Ingegneria Chimica, dei Materiali e della Produzione Industriale, University of Naples Federico II, 80125 Naples, Italy; virginia.venezia@unina.it (V.V.); giulio.pota@unina.it (G.P.); almerinda.dibenedetto@unina.it (A.D.B.); 2 Istituto Motori-CNR, 80125 Naples, Italy; v.califano@im.cnr.it; 3 Instutute for Researches on Combustion-CNR, 80125 Naples, Italy

**Keywords:** cellobiose hydrolysis, computational fluid dynamics (CFD) simulations, β-glucosidase, immobilized enzyme microreactors, monolith channel

## Abstract

The enzymatic hydrolysis of lignocellulosic biomass-derived compounds represents a valid strategy to reduce the dependence on fossil fuels, with geopolitical and environmental benefits. In particular, β-glucosidase (BG) enzyme is the bottleneck in the degradation of cellulose because it catalyzes the hydrolysis of cellobiose, a known inhibitor of the other cellulolytic enzymes. However, free enzymes are unstable, expensive and difficult to recover. For this reason, the immobilization of BG on a suitable support is crucial to improve its catalytic performance. In this paper, computational fluid dynamics (CFD) simulations were performed to test the hydrolysis reaction in a monolith channel coated by BG adsorbed on a wrinkled silica nanoparticles (WSNs) washcoat. We initially defined the physical properties of the mixture, the parameters related to kinetics and mass transfers and the initial and boundary conditions thanks to our preliminary experimental tests. Numerical simulation results have shown great similarity with the experimental ones, demonstrating the validity of this model. Following this, it was possible to explore in real time the behavior of the system, varying other specified parameters (i.e., the mixture inlet velocity or the enzymatic load on the reactor surface) without carrying out other experimental analyses.

## 1. Introduction

The exhaustion of fossil fuels and environmental pollution, particularly due to the global phenomena of atmospheric degradation (i.e., greenhouse effect, acid rains, ozone hole), led to a search for alternative, eco-sustainable and renewable energy sources such as biofuels. With respect to fossil fuels, they offer advantages in terms of global pollution and the greenhouse effect [1]. In this regard, lignocellulosic biomass has been considered a source of strategic fuel because it does not compete with food crops and it is abundant in vegetation. The main component of lignocellulosic biomass is cellulose, a polymer composed of glucose molecules which can be hydrolyzed into fermentable glucose [2].

The enzymatic hydrolysis of cellulose is carried out by the enzyme complex cellulase [3]. It is composed of endo-1,4-β-glucanases (EC 3.2.1.4), exo-1,4-β-glucanase (EC 3.2.1.91) and β-glucosidase (EC 3.2.1.21), which perform their function sequentially and synergistically [4]. Our attention has been focused on BG since it plays a crucial role in the enzymatic degradation of cellulose by hydrolyzing cellobiose to two glucose molecules. Indeed, cellobiose hydrolysis is the rate limiting factor for the whole process of the enzymatic degradation of cellulose because this species acts as an inhibitor of both endo- and exoglucanase activities [4,5].

However, free enzymes are unstable, expensive and difficult to recover, causing high costs and low production efficiency. Immobilization technology allows us to improve both the catalytic performance and thermal and operational stability of the enzyme, allowing multiple reuses of the enzyme, separation and continuous operation in industrial applications [6]. Among the techniques of enzyme immobilization, physical adsorption is the most straightforward and takes place in mild conditions so that the biocatalyst can retain its native structure and activity [7]. For this reason, we used physical immobilization to link the enzyme to an inorganic porous carrier [8].

Enzymatic hydrolysis processes are usually carried out by incubating the substrate of the reaction, cellobiose, with the free or immobilized enzyme in a batch reactor. However, a discontinuous reactor has several disadvantages related to the high consumption of the costly enzyme, idle periods in which the reactor is not operative and mass transfer limitations.

To overcome these issues, immobilized enzyme microreactors (IEMs) have proven to be a good alternative [9,10,11,12,13,14,15,16]. Microreactors allow the use of small amounts of reagents and reduce mass transfer limitations. In microfluidic reactors, reactions occur continuously and under conditions similar to those of macroscopic reactors, but they are characterized by surfaces and dimensions in terms of microns [17]. Their use is well suited to reactions that take place in a short time and which, in batch systems, are characterized by a certain inefficiency in mass and heat transfer. Additionally, the laminar flow rate occurring within the microchannels can avoid the foam formation and turbulence that often affect discontinuous reactors [10,17].

For these reasons, process intensification transforms conventional chemical processes into more economical, productive and green processes. The application of microreactors to enzyme catalyzed reactions has shown very good results in terms of reaction times and performance with respect to batch processes [11].

Recently, we realized the intensification and the engineering of the enzymatic hydrolysis process through the transition from a batch reactor to a plug-flow microreactor by the application of ceramic cordierite monoliths whose washcoat consisted of β-glucosidase immobilized into a mesoporous silica nanoparticle support [18].This inorganic matrix can be used for many applications such as drug delivery and fluorescence biological probes and can be an efficient support for enzyme adsorption [19,20,21,22]. Silica nanoparticles have high chemical, mechanical and thermal stability. In our previous works, we used Wrinkled silica nanoparticles (WSNs), whose peculiar morphology created a favorable microenvironment for catalysis [8,23,24,25]. We showed the great potential of the microreactor with respect to batch, leading to higher conversion in lower reaction times. The use of an enzyme-loaded honeycomb monolith within a microfluidic reactor can improve reaction efficiency and facilitate continuous operation, reuse and regeneration [16,17,18,19,20,21,22,23,24,25,26].

In order to properly design the microreactor and to optimize the performance, full investigations of the role of the operating conditions are required. In this context, mathematical modeling plays a crucial role in the development and design of chemical (micro) reactors.

To this end, computational fluid dynamics (CFD) models have been extensively used [27,28,29]. In this work, we developed a CFD model of the IEMs to simulate the effect of temperature reaction, mixture inlet velocity and enzymatic load on the WSNs washcoat on the reaction performance. The model results will also support the experimental activity to optimize the IEMs operation.

## 2. Model Description

In our CFD simulations, a two-dimensional axisymmetric model has been developed to simulate the behavior of the enzymatic reaction in a single channel of cordierite monolith [30].

**Figure 1:**
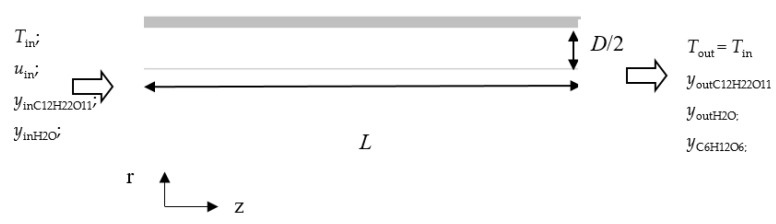
Scheme of a cordierite channel.

In Figure 1, the channel of monolith (*D* = 1 mm, *L* = 10 mm) is shown.

It has been assumed that the enzyme was uniformly deposited onto the walls of the channel.

Since the Reynolds number is around 10^−2^, reactive flow in the channel is laminar, avoiding the foam formation and turbulence often affecting batch reactors.

The equations for conservation of total mass, momentum, energy and chemical species are solved in liquid phase and are coupled to the Stokes equations.

Continuity equation:

(1) $$\frac{\partial \left(\rho u\right)}{\partial z}+\frac{1}{r}\;\frac{\partial r\left(\rho v\right)}{\partial r}$$

Momentum equations:

(2) $$\frac{\partial \left(\rho uu\right)}{\partial z}+\frac{1}{r}\;\frac{\partial r\rho vu}{\partial r}=\;-\frac{\partial p}{\partial z}+\frac{1}{r}\;\frac{\partial r\tau_{zr}}{\partial r}+\frac{\partial \tau_{zz}}{\partial z}$$

(3) $$\frac{\partial \rho uv}{\partial z}+\frac{1}{r}\;\frac{\partial r\rho vv}{\partial r}=\;-\frac{\partial p}{\partial r}+\frac{1}{r}\frac{\partial r\tau_{rr}}{\partial r}+\frac{\partial \tau_{zr}}{\partial z}$$

In the above equations, ρ is the density of the mixture, *v* and *u* are the radial and axial component of the flow velocity of the fluid, respectively; *r* and *z* are the radial and axial coordinates in the system, respectively; τ is the stress tensor working on the liquid mixture, and *p* is the pressure of the fluid.

Catalytic experimental tests were run isothermally. However, the current reaction temperature can change due to the reaction, which is weakly exothermic. To preliminarily quantify the reaction thermicity, we calculated the adiabatic temperature increase (Δ*T_ad_*) according Equation (4):

(4) $$\Delta T_{ad}=-\frac{C_{C}\cdot \Delta H_{r}}{c_{p}\cdot \rho }$$

where *C_C_* is the cellobiose concentration in the starting solution ($\mathrm{mol}/m^{3}$), Δ*H_r_* is reaction heat (kJ/mol), *c_p_* is the specific heat of the reacting mixture (kJ/(kg∗K)), and ρ is the density of the reacting mixture ($\frac{\mathrm{kg}}{m^{3}}$).

The reaction heat has been calculated as follows:

(5) $$\Delta H_{r}=\Sigma \nu_{i}\Delta H_{i}$$

where *ν_i_* is the stoichiometric coefficient (positive for the products and negative for the reagents) and Δ*H_i_* is the heat of formation (kJ/mol). The reaction is as follows:

$$C_{12}H_{22}O_{11}+H_{2}O\to 2C_{6}H_{12}O_{6}$$

Table 1 shows the values of the parameters used to calculate the Δ*T_ad_*.

The value of Δ*T_ad_* is equal to 3.5 K. Therefore, we can consider the reactor isothermal.

Chemical species balance in the liquid phase:

(6) $$\frac{\partial \rho uy_{i}}{\partial z}+\frac{1}{r}\frac{\partial r\rho vy_{i\;}}{\partial r}=\frac{\partial }{\partial z}\left(J_{z,i}\right)+\frac{1}{r}\frac{\partial }{\partial r}\left(rJ_{r,i}\right)+S*r\;\;\;\;i=1,\ldots ,N_{s}-1$$

where *y_i_* is the mass fraction, *J_z,i_* and *J_r,i_* are the axial and radial components of the diffusion velocity of the *i*-th liquid species, while r is the reaction rate, respectively. The latest species mass fraction is computed as one minus the sum of the other species mass fractions.

The boundary conditions are listed in the following.

At the inlet, we assumed the Dirichlet type boundary conditions.

(7) $$@\;z\;=\;0\;\;\;\mathrm{and}\;\;\;0\;<\;r\;<\;\frac{D}{2}:\;\;\;\;\;\;\;u\;=\;u_{in},\;\;\;v\;=\;0,\;\;y_{i}\;=\;y_{i,in}\;@\;z\;=\;L\;\;\;\mathrm{and}\;\;\;0\;<\;r\;<\;\frac{D}{2}:$$

(8) $$µ\left(\frac{\partial u}{\partial r}+\frac{\partial v}{\partial z}\right)=0$$

(9) $$2\;µ\frac{\partial u}{\partial z}\;=\;p\;-\;p_{\mathrm{atm}}$$

(10) $$\frac{\partial y_{i}}{\partial z}$$

where µ is the dynamic viscosity of the mixture.

(11) $$@\;0<z\;<\;L\;\mathrm{and}\;r\;=\;0:\;\;\;\;\frac{\partial u}{\partial r}\;=\;v\;=\;\frac{\partial y_{i}}{\partial r}\;=\;0;$$

(12) $$@\;0<z\;<\;L\;\mathrm{and}\;r\;=\;\frac{D}{2}:\;\;\;u\;=\;v\;=\;0,\;\mathrm{Surface}\;\mathrm{Area}\;\mathrm{Washcoat}\;\mathrm{Factor};$$

The surface area washcoat factor (*SF*) refers to the catalyst/support mass ratio, according to the following equation:

(13) $$SF=\;\frac{\;m_{\mathrm{adsorbed}\;\mathrm{enzyme}}}{m_{\mathrm{support}}}$$

where *m*_adsorbed enzyme_ is the amount of immobilized enzyme on the wall surface of the monolith, while *m*_support_ is the amount of WSNs deposited on a single monolith. This parameter was assessed through our previous experimental analysis [18].

The enzymatic kinetics followed a Michaelis−Menten behavior. The simulations were carried out by choosing a very low inlet cellobiose concentration. Since the experimental conditions have proven that cellobiose concentrations higher than 0.01 kmol/m^3^ can inhibit β-glucosidase, reactants and products, in this case, it is possible to consider a first order reaction. The value of the kinetic constants was derived by fitting our experimental data [18].

In particular,

*K*_0_ = 2.0 × 10^9^ $\frac{1}{s}$

*E*_a_ = 6.65 × 10^7^ J/kmol

The stoichiometric coefficient and the rate exponent are given in Table 2.

The mixture molecular viscosity is considered equal to that of water (5.5 × 10^−4^ kg/(m∗s)) since the solution is very diluted, while species diffusivity in aqueous media was 5 × 10^−6^ $m^{2}/s$.

The model equations are discretized using a finite volume formulation on a structured mesh built by means of the Design Modeler and Meshing packages by Ansys (Release 19.0).

Mesh parameters are reported in Table 3.

The spatial discretization of the model equations uses second order schemes for all terms, except for the momentum term that is treated with a first order central difference scheme.

Computations were performed by means of the segregated solver of the Fluent ANSYS code, adopting the SIMPLE method for treating the pressure-velocity coupling.

Inlet temperature, velocities and liquid compositions are specified as the feed conditions.

Table 4 summarizes the inlet parameter values.

Some CFD simulations were carried out on the basis of the following experimental data in order to obtain the validation with the experimental results [18]:

*T* = 50 °C, 60 °C or 70 °C

*SF* = 0.15

Molar concentration of cellobiose = 8.4 × 10^−5^

*K*_0_ = 2.0 × 10^9^ $\frac{1}{s}$

*E*_a_ = 6.65 × 10^7^ J/kmol

Inlet mixture velocity: 1.3 × 10^−5^ m/s

In our experiments [18], the flow rate entering the reactor flows through a 1-inch tube, resulting in a flat velocity profile at the inlet of the monolithic reactor. Our experimental inlet liquid velocity (1.3 × 10^−5^ $\frac{m}{s}$) was evaluated by dividing the flow rate at the inlet of the monolith by the cross-section of the same, while the cellobiose molar fraction was estimated while taking into account that its inlet concentration was 0.00467 kmol/m^3^ [5,8,18,23].

## 3. Results and Discussion

### 3.1. Effect of Temperature

In the following paragraph, the base case (*T* = 50 °C) is first discussed. The conversion of cellobiose to glucose was determined according to the following relation:

(14) $$x=\frac{Ccin-Ccout}{Ccin}*100$$

where *c_cin_* and *c_cout_* are the inlet and the outlet cellobiose concentration, respectively.

**Figure 2:**
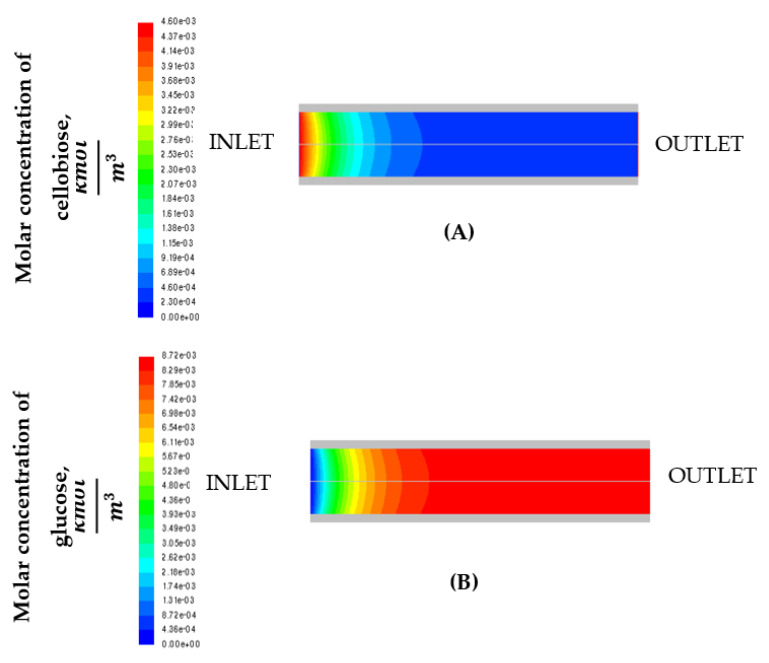
Contours of molar concentration of cellobiose (**A**) and molar concentration of glucose (**B**).

Figure 2 shows the contours of the molar concentration of cellobiose (A) and of the molar concentration of glucose (B) in the cordierite channel.

From Figure 1, it turns out that concentration variations are significant in the axial direction rather than in the radial direction, suggesting a plug flow reactor (PFR) like behavior.

**Figure 3:**
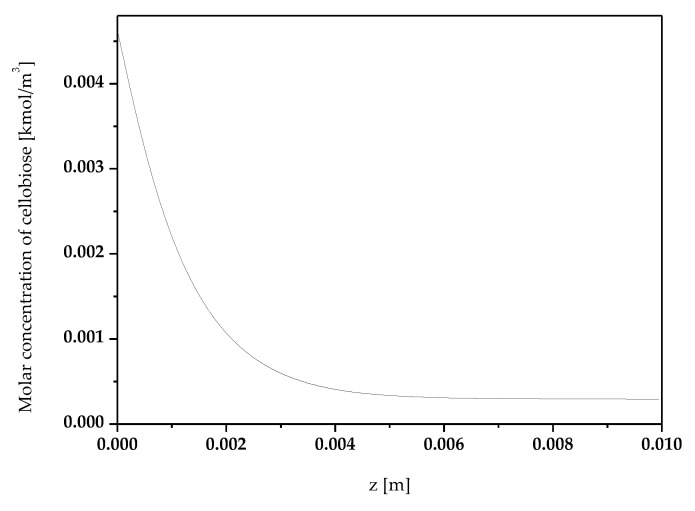
Molar concentration of cellobiose (kmol/m^3^) along the axial coordinate z.

Graph of Figure 3 shows the molar concentration of cellobiose (kmol/m^3^) along the axial coordinate z.

**Figure 4:**
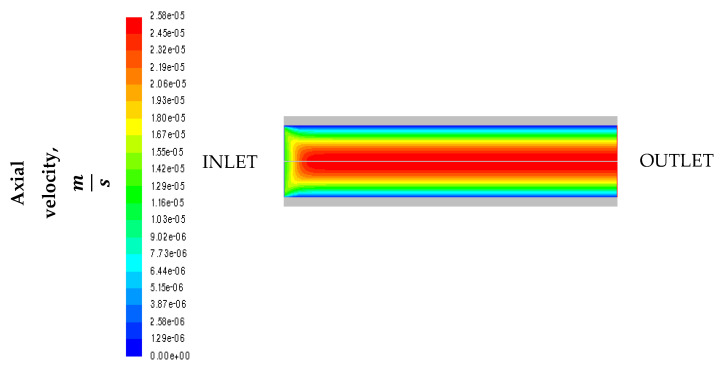
Trend of the axial velocity.

Figure 4 shows the trend of the axial velocity in the cordierite channel. The flow is laminar and the parabolic profile is found: the mixture velocity is maximum along the channel axis and decreases approaching the wall since the fluid adheres to the walls (no slip condition).

**Figure 5:**
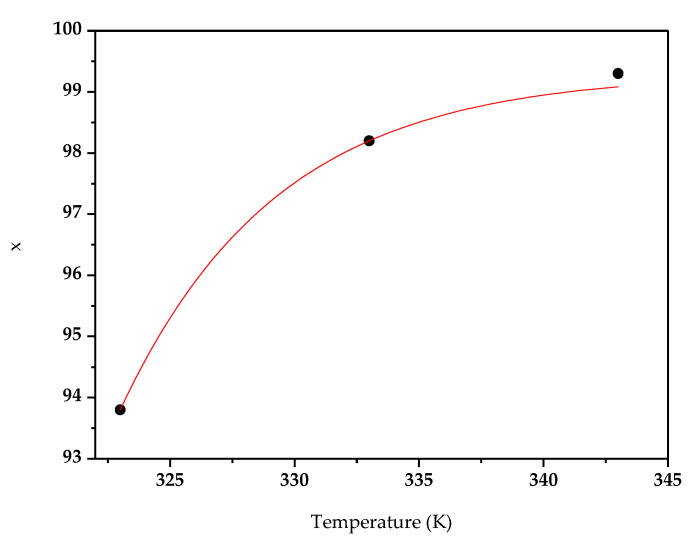
Cellobiose conversion x (%) vs. temperature, K.

Simulations were run at varying temperatures up to 70 °C. Figure 5 shows the cellobiose conversion x as a function of temperature.

The continuous lines originate from a nonlinear fit of the numerical results, reported by the dots.

A Langmuir function has been used to fit cellobiose conversion vs. temperature. On increasing the temperature up to 70 °C, the cellobiose conversion increases from around 94% up to around 100%. The system is under kinetic control and cellobiose conversion increases by increasing the temperature. In favor of this, our experimental tests highlighted that the enzyme retained its native structure until 70 °C thanks to the immobilization process [18,23,31]. However, further increase of temperature caused the denaturation of the enzyme, leading to the fast deactivation of the catalyst.

### 3.2. Effect of Enzyme Immobilization Yield

In our experimental tests [5,8,18], the adsorption of the enzyme on the structured substrates was carried out in a buffer solution at pH 5 containing 0.03 mM of BG and 2 mg/mL of WSNs. The mixture was kept under gentle stirring overnight at room temperature. Then, the biocatalyst obtained was suspended in a new buffer solution at pH 5 for 10 min to remove the non-adsorbed enzyme. The amount of immobilized enzyme was equal to 6 mg for 40 mg of support.

Numerical simulations were performed at various catalyst loadings on each channel of the microreactor, taking as reference the *SF* corresponding to the experimental tests (*SF* = 0.15).

**Figure 6:**
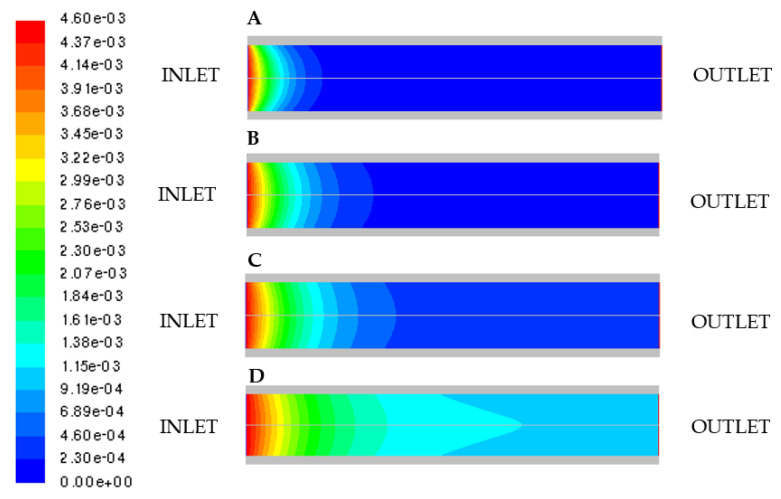
Molar concentration of cellobiose by varying the adsorbed enzyme mass: (**A**) *SF* = 0.5; (**B**) *SF* = 0.15; (**C**) *SF* = 0.075; (**D**) *SF* = 0.0375.

In Figure 6, the molar concentration of cellobiose is shown, as obtained at different values of *SF*.

A smaller amount of adsorbed enzyme (Figure 6C,D) results in lower outlet conversion.

It was found that, on decreasing *SF*, the cellobiose conversion significantly decreases. In particular, conversion goes from 99.6 % at *SF* = 0.5 (case A) to 75.7% when lowering *SF* to 0.0375 (case D).

**Figure 7:**
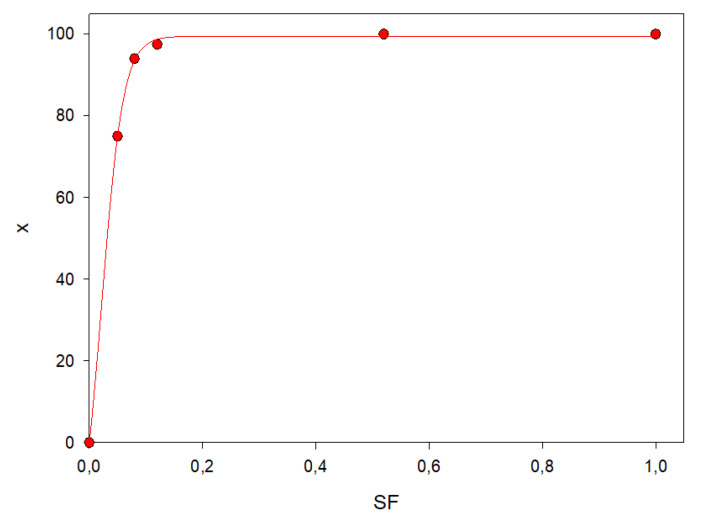
Cellobiose conversion x (%) over *SF*.

Figure 7 shows the cellobiose conversion x over the *SF* factor.

The continuous lines originate from the nonlinear fit of the numerical results, reported by the dots.

A Michaelis–Menten curve has been considered the most appropriate to describe both cellobiose conversion and *SF*. When the *SF* factor is 0.075, the conversion reaches a plateau, corresponding to the complete conversion of cellobiose. Therefore, an increase in the amount of the adsorbed biocatalyst is useless to improve the final glucose concentration and would only cause extra costs. Consequently, we proved that a very low amount of immobilized enzyme can maximize the glucose production.

### 3.3. Effect of Mixture Inlet Velocity

In batch reactors, the mass transport of the substrate from the bulk of the reaction medium to the active site of the enzyme significantly affects the reaction and, therefore, cellobiose conversion. To prove that, in a continuous reactor, mass transfer is not limiting, we performed simulations by changing the inlet liquid velocity.

**Figure 8:**
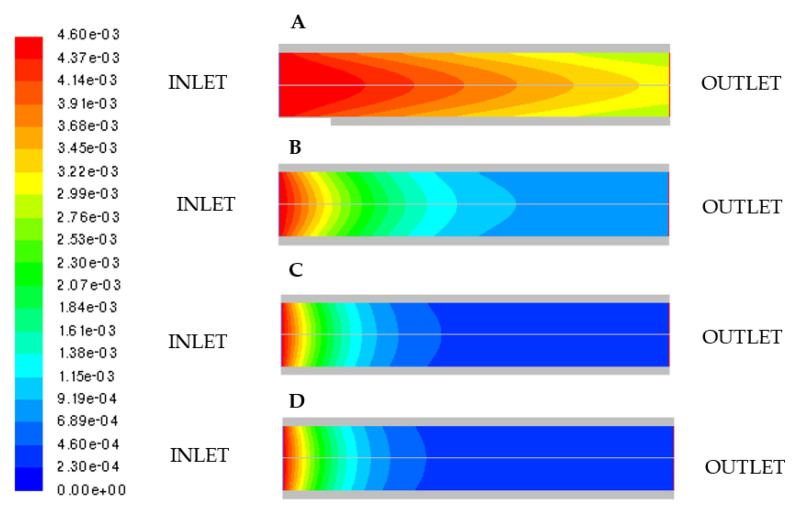
Molar concentration of cellobiose by varying mixture inlet velocity. (**A**) *u_in_* = 0.1297 $\frac{m}{s}$, (**B**) *u_in_* = 1.297 × 10^−2^ $\frac{m}{s}$, (**C**) *u_in_* = 1.297 × 10^−3^ $\frac{m}{s}$, (**D**) *u_in_* = 1.297 × 10^−5^ $\frac{m}{s}$.

In Figure 8, the molar concentration of cellobiose obtained at four values of the inlet liquid velocity is shown.

Cellobiose conversion decreases on increasing *u_in_*. As a consequence, the reaction rate is controlled by intrinsic kinetics and it is mainly affected by the residence time.

This result was highlighted as evaluating the cellobiose conversion for different values of Damköler number (*Da*) [28], which is defined as follows:

(15) $$Da=\frac{\mathrm{flow}\;\mathrm{time}\;\mathrm{scale}}{\mathrm{chemical}\;\mathrm{time}\;\mathrm{scale}}$$

For a first order reaction, *Da* follows this relation:

(16) Da=k∗τ

where *k* is the Arrhenius constant and τ is the residence time of the liquid mixture in the channel of the microreactor. In particular, τ is expressed as follows:

(17) $$\tau =\frac{V}{Q}$$

where *V* is the volume of a single channel (m^3^), while *Q* is the volumetric flow rate (m^3^/s) at the inlet of the channel.

**Figure 9:**
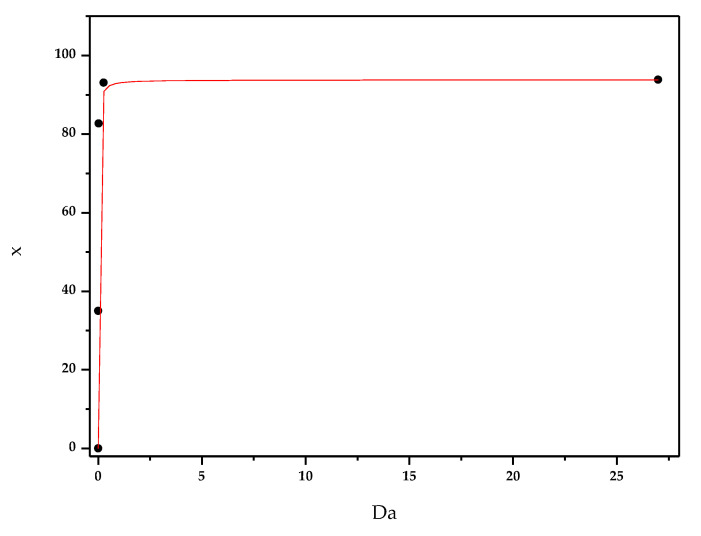
Cellobiose conversion x (%) vs. Da.

Since both the section and the length of microchannel were fixed, we expressed the variation of cellobiose conversion with *u_in_* by plotting x vs. Da, as shown in Figure 9.

The continuous lines originate from a nonlinear fit of the numerical results, reported by the dots.

A Michaelis–Menten curve was considered the most appropriate to describe cellobiose conversion vs. Da. We can observe that, for values of Da higher than 0.27, x approaches the asymptotic value of around 94%; on the other hand, lowering Da below 0.27, cellobiose conversion decreases dramatically. This happens because higher values of Da number correspond to lower values of u_in_ and, therefore, higher values of τ, favoring complete conversion.

## 4. Conclusions

Enzymatic hydrolysis of cellobiose to glucose in a microchannel reactor was investigated through CFD simulations. In order to evaluate the performance of this system, the effects of three different variables of interest were investigated:

- The temperature of the reaction;
- The catalyst loading by evaluating the *SF* factor;
- The mixture inlet velocity.

The results prove that the reaction is under kinetic control and that maximum conversion is reached at 70 °C. Moreover, this evidence was confirmed by our preliminary experimental tests since immobilized BG retains its maximum activity at temperatures up to 70 °C.

Numerical simulations were performed at various catalyst loadings *SF* on the inner surface of the microreactor. The results showed that a higher amount of adsorbed enzyme led to higher outlet conversion. In particular, the conversion goes from 75.7% at *SF* = 0.0375 to 99.6% at *SF* = 0.5.

Cellobiose conversion increases if the mixture inlet velocity decreases. As a consequence, the reaction rate is controlled by intrinsic kinetics and it is mainly affected by the residence time rather than by diffusional limitations.

These aspects were evaluated by taking into account the Da number: when the adimensional number is greater than 0.27, complete cellobiose conversion is accomplished.

Finally, result simulations showed great affinity with experimental ones, demonstrating the validity of this model. Other simulations were carried out to estimate cellobiose conversion under different inlet conditions (i.e., mixture inlet velocity variation), without carrying out further experimental analyses.

## Figures and Tables

**Table 1:** Parameters used to calculate the Δ*T*_ad_.

	Δ*H_i_*, kJ/mol	*ν_i_*	*c*, mol/m^3^	*c_p_*, (kJ/(kg*K)	*ρ*, kg/m^3^
**Cellobiose**	−5401.50	−1	4.67		
**Water**	−285.85	−1			
**Glucose**	−1273.30	2			
**Mixture**				4.186	1000

**Table 2:** Stoichiometric coefficient and rate exponent.

Species	Stoichiometric Coefficients	Rate Exponent
**Cellobiose**	1	1
**H_2_O**	1	0
**Glucose**	2	0

**Table 3:** Mesh parameters.

Mesh Parameters	Number
**Cells**	12,006
**Faces**	30,029
**Nodes**	12,692
**Partitions**	1

**Table 4:** Inlet parameter values.

Parameter	Value
**Mixture inlet temperature, *T_in_*** (**K**)	323–343
**Mixture inlet velocity, *u_in_*** (**m s^−1^**)	0.13; 0.013; 1.3 × 10^−3^; 1.3 × 10^−5^
**Inlet C_12_H_22_O_11_ molar fraction**	8.4 × 10^−5^
**Surface area washcoat factor**	0.0375–0.075–0.15–0.5
